# Bayesian one‐step IPD network meta‐analysis of time‐to‐event data using Royston‐Parmar models

**DOI:** 10.1002/jrsm.1253

**Published:** 2017-07-25

**Authors:** Suzanne C. Freeman, James R. Carpenter

**Affiliations:** ^1^ MRC Clinical Trials Unit at UCL Aviation House 125 Kingsway London WC2B 6NH UK; ^2^ Department of Health Sciences Univeristy of Leicester, University Road Leicester LE1 7RH UK; ^3^ London School of Hygiene & Tropical Medicine Keppel Street London WC1E 7HT UK

**Keywords:** IPD, network meta‐analysis, Royston‐Parmar, time‐to‐event data

## Abstract

Network meta‐analysis (NMA) combines direct and indirect evidence from trials to calculate and rank treatment estimates. While modelling approaches for continuous and binary outcomes are relatively well developed, less work has been done with time‐to‐event outcomes. Such outcomes are usually analysed using Cox proportional hazard (PH) models. However, in oncology with longer follow‐up time, and time‐dependent effects of targeted treatments, this may no longer be appropriate. Network meta‐analysis conducted in the Bayesian setting has been increasing in popularity. However, fitting the Cox model is computationally intensive, making it unsuitable for many datasets. Royston‐Parmar models are a flexible alternative that can accommodate time‐dependent effects. Motivated by individual participant data (IPD) from 37 cervical cancer trials (5922 women) comparing surgery, radiotherapy, and chemotherapy, this paper develops an IPD Royston‐Parmar Bayesian NMA model for overall survival. We give WinBUGS code for the model. We show how including a treatment‐ln(time) interaction can be used to conduct a global test for PH, illustrate how to test for consistency of direct and indirect evidence, and assess within‐design heterogeneity. Our approach provides a computationally practical, flexible Bayesian approach to NMA of IPD survival data, which readily extends to include additional complexities, such as non‐PH, increasingly found in oncology trials.

## 1 INTRODUCTION

Network meta‐analysis (NMA) is the extension of pairwise meta‐analysis (MA) to a network of clinical trials in which each trial compares at least 2 treatments from a set of treatments in a specific disease area. Network meta‐analysis uses a single statistical model to combine both direct and indirect evidence from all of the trials in a network to calculate treatment effect estimates for every treatment comparison, regardless of whether 2 treatments have been compared directly, and thus permits ranking of the treatments.

Most NMA methods have developed as a result of extending MA methods for 2 treatments to 3 or more treatments to take advantage of the indirect evidence. Modelling approaches for continuous and binary outcomes are relatively well developed, but less work has been done with time‐to‐event outcomes. Such outcomes have usually been analysed using semiparametric Cox proportional hazard (PH) models,^1^ but in oncology with longer follow‐up of trials, and time‐dependent effects of targeted treatments, we are seeing increasing evidence of non‐PH so this may no longer be appropriate.^2^, ^3^

Network meta‐analysis can be conducted using individual participant data (IPD) or aggregate data (AD) with IPD considered the gold standard for both MA and NMA.^4^ Individual participant data allows trials to be re‐analysed in a consistent manner standardising inclusion and exclusion criteria, re‐coding covariates, including previously excluded patients and using up‐to‐date follow‐up information. Data can be checked against the published results to ensure the quality of randomisation and follow‐up.^4^ Most importantly, IPD provides greater statistical power for subgroup analyses, enables the analysis of patient level covariates, and is essential for investigating interactions between treatment and patient level covariates.^5^, ^6^

Network meta‐analysis conducted in the Bayesian setting has been increasing in popularity in recent years.^7^ The Bayesian framework naturally handles random effects, avoiding awkward numerical integration. In particular, Crowther et al^8^ reported that—when the number of trials in the MA is small—maximum likelihood tends to underestimate random effect variances, and this issue is alleviated with a Bayesian analysis (albeit at the expense of some overestimation of the variances). Other attractions include ready inference for treatments never compared directly, easy assessment of network consistency, a natural ranking method, which allows calculation of cumulative rankings to determine the probability of a treatment being 1 of the top 3 most effective treatments, and the ability to adjust for correlations that arise from the inclusion of multiarm trials.^9^, ^10^, ^11^ Another potential advantage of the Bayesian approach is that, if we wish to extend the models by adjusting for patient level covariates, then a Bayesian model can readily incorporate the imputation of any missing values (Carpenter and Kenward^12^, p. 47). Bayesian inference also provides a natural framework for prediction.^13^

Bayesian NMA models are commonly fitted in WinBUGS. However, fitting the Cox PH model in the Bayesian setting is computationally intensive, as each individual's data have to be repeated for each risk set they belong to. This makes it extremely cumbersome even for moderately sized datasets, such as our motivating cervical cancer data described below. Therefore, alternative methods for time‐to‐event data are needed.

Crowther proposed 2 alternatives to the Cox model for time‐to‐event outcomes, which could be used for MA.^8^, ^14^ First, a one‐step IPD MA using a Poisson generalised linear model (GLM), which could be implemented with fixed or random effects and with baseline hazard stratified by trial. This model was extended to include treatment‐covariate interactions and to allow non‐PH of the treatment effects.^14^ Crowther et al^14^ demonstrated the use of Poisson GLM in both the frequentist and Bayesian settings. To fit a Poisson GLM with time‐to‐event data, the time scale must be split into intervals.^14^ A substantial number of intervals may be required in applications, and their location and length may be important.^15^ ^(p65)^ Royston and Lambert further comment (p90) on the potential computational issues with the piecewise exponential approach with large datasets. For example, when fitting the piecewise hazard model in WinBUGS, the data for patients in the risk set at the beginning of each interval need to be repeated. Assessing and modelling non‐PH is also relatively complex using this approach relative to a spline‐based approach (see Subsection 4.1.1).

By contrast, Royston and Lambert (p78) find that (provided the log cumulative hazard is modelled) the precise knot location is relatively unimportant. These points, alongside the flexibility of splines, motivated Crowther et al^8^ to develop maximum likelihood approaches for random effect models with splines, using Gauss‐Hermite quadrature for the numerical integration. However, maximum likelihood methods become increasingly challenging as the number of random effects increase and may also struggle when the number of trials in an MA is small.^8^, ^15^

In this context, Jansen^16^, ^17^ explored using fractional polynomials^18^ to model the baseline hazard in two‐step random effects NMA of IPD time‐to‐event data. This work and the work of Ouwens^19^ were extended to include treatment‐covariate interactions, which allowed the model to adjust for confounding.^17^ However, fractional polynomials can result in unexpected end effects. Specifically, the shape of a fractional polynomial at each end of the dataset, where there is often less information, may be unduly influenced by what happens in the middle of the dataset.

In the light of this, we concluded that the Royston‐Parmar model,^20^ with the baseline log‐cumulative hazard modelled by restricted cubic splines (RCSs), is a natural way forward. The complexity and flexibility of the model are determined by the degrees of freedom of the RCS. Restricted cubic splines have the advantage over fractional polynomials that they are linear at each end, and so reduce the possibility of undesirable end effects. They are therefore more likely to provide a flexible yet robust approach, appropriate for trials in most networks.

Further, as we show below, such models can be readily fitted in WinBUGS,^21^ but without the need to expand the data. For networks containing many thousands of patients, this is a key practical advantage.

Therefore, in this paper, we bring together the flexibility of Bayesian modelling and the Royston‐Parmar model, describing a one‐step IPD NMA of time‐to‐event data to a network of clinical trials in cervical cancer. We show how including a treatment‐ln(time) interaction can be used to conduct a global test for PH, illustrate how we can test for consistency of direct and indirect evidence, and assess within design heterogeneity (ie, heterogeneity between trials of the same design). We give commented WinBUGS code for fitting the model. Network meta‐analysis combines direct randomised evidence with indirect evidence, and this combination essentially relies on the external validity of the direct evidence. When presenting the results, we therefore propose and illustrate, presenting the direct and indirect treatment estimates alongside the combined estimate.

The paper is structured as follows. We start by describing our dataset in Section 2. In Section 3, we review the Royston‐Parmar model and apply it to the MA setting before extending it to the NMA setting in Section 4. In Section 5, the Royston‐Parmar NMA model is applied to the cervical cancer dataset with annotated code implementing our approach provided in Appendix A. We conclude with a discussion in Section 6.

## 2 CERVICAL CANCER DATA

**Figure 1:**
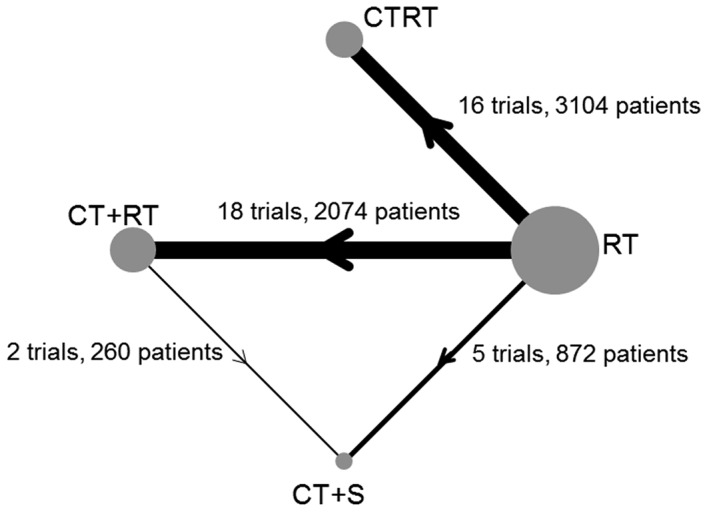
Cervical cancer network diagram. Node size and edge thickness are proportional to the number of studies involved in each direct comparison. RT, radiotherapy; CTRT, chemoradiation, CT+RT, neoadjuvant chemotherapy plus radiotherapy; CT+S, neoadjuvant chemotherapy plus surgery. NB: the numbers for each treatment arm do not add up to the total number of patients included in the network as multiarm patients are counted twice. There are a total of 37 trials in this network; however, in the figure, the 2 multiarm trials are counted 3 times each as they are included in the number of trials for each pairwise comparison. Arrows denote direction of treatment comparison in NMA models (see Section 4.1)

Our motivating data come from 3 meta‐analyses of randomised controlled trials (RCTs) in cervical cancer performed by the Chemoradiotherapy for Cervical Cancer Meta‐Analysis Collaboration^22^ and the Neoadjuvant Chemotherapy for Cervical Cancer Meta‐Analysis Collaboration.^23^ The 3 meta‐analyses considered 4 different treatments: radiotherapy (RT), chemoradiation (CTRT), neoadjuvant chemotherapy plus radiotherapy (CT+RT), and neoadjuvant chemotherapy plus surgery (CT+S) using 4 different designs: RT vs CTRT (18 trials), RT vs CT+RT (16 trials), RT vs CT+S (5 trials), and RT vs CT+RT vs CT+S (2 trials) (Figure 1). The Neoadjuvant Chemotherapy for Cervical Cancer Meta‐Analysis Collaboration^23^ conducted one systematic review to consider 2 related but separate treatment comparisons: RT vs CT+RT and RT vs CT+S. Trial accrual periods ranged from 1982 to 1999. The Chemoradiotherapy for Cervical Cancer Meta‐Analysis Collaboration^22^ conducted one systematic review to compare RT and CTRT. Trial accrual periods ranged from 1987 to 2006. Both systematic reviews were completed following detailed prespecified protocols.

The RT vs CTRT comparison included a total of 18 RCTs and 4818 patients. In the original publication, 5 of these trials were excluded from the main analysis, as patients on at least one of the treatment arms received additional treatment. This resulted in a subset of 13 trials (3104 patients), which were identified and used for the main analysis. Within this subset of 13 trials 2 three‐arm trials combined 2 different forms of CTRT and compared them with a single control arm and 3 4‐arm trials were split into 2 unconfounded comparisons of RT vs CTRT for analysis as separate trials. This resulted in 16 trials included in the main analysis. As in the original publication, the data will be treated in the same way throughout this paper.

Across the 3 meta‐analyses that form our network of trials, overall survival data were available for 5922 patients from 37 RCTs (35 two‐arm RCTs, 2 three‐arm RCTs).

## 3 REVIEW OF THE ROYSTON‐PARMAR MODEL AND IMPLEMENTATION OF PAIRWISE META‐ANALYSIS METHODS

### 3.1 Royston‐Parmar model for the log cumulative hazard rate

To implement the Royston‐Parmar model in the NMA setting, we use an RCS to model the log baseline cumulative hazard rate for each trial. An RCS is a piecewise polynomial with additional constraints to ensure a smooth log baseline cumulative hazard. An RCS has a number of interior knots as well as boundary knots at the minimum and maximum of the uncensored survival times. The fitted RCS is continuous, has continuous first and second derivatives, and is forced to be linear before the first knot and after the last knot.^24^ Further details on RCS can be found in Lambert and Royston,^24^ Royston and Parmar,^20^ and Royston and Lambert.^15^

The spline function for patient *i* in trial *j* with *p* interior knots can be written as

(1) $$\begin{array}{ccc} s_{j}(\ln(t_{i})) & =\gamma_{1}+\gamma_{2}u_{0}(\ln(t_{i})) & \\ & \;+\gamma_{3}u_{1}(\ln(t_{i}))+\text{⋯}+\gamma_{p+2}u_{p}(\ln(t_{i})), \end{array}$$

where $\ln(t_{i})$ is the natural logarithm of event time for patient *i*, $u_{0}(\ln(t_{i})),u_{1}(\ln(t_{i})),\text{⋯},u_{p}(\ln(t_{i}))$ are the orthogonalised basis functions and the *γ*'s their coefficients. Basis functions are defined in Appendix A.

The RCS for the log cumulative hazard can be incorporated into a PH flexible parametric model with *x*_*i*_ the treatment indicator for patient *i* and *β* the coefficient,

(2) $$log\{H(t|x_{i})\}=\eta_{ij}=s_{j}(\ln(t_{i}))+\beta x_{i}.$$

Covariates can also be included in (2) as adjustment factors if necessary. To fit this flexible parametric model (2), the log likelihood of the observed data must be calculated. To derive the log likelihood, the derivative of *η*_*ij*_ is required,

(3) $$d\eta_{ij}=\gamma_{2}du_{0}(\ln(t_{i}))+\gamma_{3}du_{1}(\ln(t_{i}))+\text{⋯}+\gamma_{p+2}du_{p}(\ln(t_{i})),$$

where d*u*_*p*_ is the derivative with respect to $\ln(t_{i})$ of *u*_*p*_.

The likelihood *l*_*ij*_ for patient *i* is then

(4) $$log(l_{ij})=\left\{\begin{array}{cc} log(d\eta_{ij})+\eta_{ij}-\exp(\eta_{ij}) & \text{for an observed event,}\\ -\exp(\eta_{ij}) & \text{for a censored observation.} \end{array}\right.$$

WinBUGS can be used for Bayesian inference with this likelihood. WinBUGS does not have an appropriate inbuilt distribution for the Royston‐Parmar model; therefore, the “zeros trick” is required to enable a general likelihood to be specified.^15^ The probability density function of the Poisson distribution is $f(y|\lambda )=\frac{\lambda^{y}\exp(-\lambda )}{y!}$. The “zeros trick” works because when *y* is set equal to zero, the Poisson likelihood is exp(−λ). Therefore, if we set *λ* equal to the negative log likelihood contribution for each patient and we use a psuedo observation “*y*=0” for each patient, using a Poisson model gives us the correct likelihood.^15^ As a Bayesian approach, WinBUGS has the added advantage of the flexibility to extend models (eg, to include multiple random effects and covariates) without involving numerical integration. Then, the fixed effect of treatment in (2) can be readily replaced by a random effect if desired.

#### 3.1.1 Testing for non‐PHs

Non‐PH can be assessed by including a treatment‐ln(time) interaction in (2):

(5) $$\ln\{H(t|x_{ij})\}=s_{j}(\ln(t_{i}))+\beta x_{i}+\alpha x_{i}\left(\ln\left(t_{i}\right)\right),$$

where $x_{i}\left(\ln\left(t_{i}\right)\right)$ is the treatment‐ln(time) interaction term for patient *i* and *α* the coefficient. In (3), the derivative of (2) is calculated with respect to ln(t); therefore, (3) must be updated appropriately when we include treatment‐ln(time) interactions. A further extension is to allow *α* to be random across (groups of) trials; see Section 4.1.1. If the treatment‐ln(time) interaction term is statistically significant, then there is evidence of non‐PH in the pairwise comparison. If this is the case, then a treatment‐ln(time) interaction for this pairwise comparison should be included in the NMA model.

Before conducting MA or NMA, each trial should be assessed individually for evidence of non‐PH. A natural way to do this is to calculate the Schoenfeld residuals, which can be examined graphically and formally tested for nonproportionality using a *χ* ^2^ test. As each trial is independent of each other, in each MA, if desired, we can add up the values of the *χ* ^2^ statistics to provide an overall nonspecific test with degrees of freedom equal to the number of trials in the MA.

The Schoenfeld residual test, applied to each trial in turn, looks for any evidence of a different trend in the Schoenfeld residuals between the treatment groups.^25^ It highlights any trials that show a marked departure from PH, which should be investigated further before including the trial in a PH NMA. Such departures may be due to quirks of the design or follow‐up. By contrast, testing the null hypothesis that *α*=0 in (5) provides a more powerful test of the specific hypothesis that the log‐cumulative hazard has a different linear trend in log(t) in the different treatment groups. If, across the (N)MA, we reject *α*=0, then summarising treatment effects by a single hazard ratio is inappropriate.

### 3.2 Estimation

To fit the Royston‐Parmar model in WinBUGS, the basis functions for the RCS must be calculated and then orthogonalised using Gram‐Schmidt orthogonalisation. The basis functions can be calculated in Stata or any other statistical software package. Full details on this process are provided in Appendix A and Lambert and Royston.^24^

Once calculated, the basis functions are passed to WinBUGS to fit the one‐step NMA model (6) in which the logarithm of the baseline cumulative hazard function is modelled as a “natural” cubic spline function of log time.^20^ The default knot locations for RCS are based on centiles of the uncensored survival times with additional boundary knots placed at the minimum and maximum values of the uncensored survival times. Royston and Lambert do not recommend models with more than 3 knots, as the resulting curves can be unstable; however, they do acknowledge that in larger datasets a larger number of knots may be required.^15^ It has been shown recently that parameter estimates are generally robust to knot locations^26^; however, it is also possible to choose knot locations. With the cervical cancer data, we chose our own knot locations because we wanted to ensure that the log cumulative hazard resulting from the WinBUGS model was as similar to the nonparametric Nelson‐Aalen estimate of the log cumulative hazard as possible for each trial. Starting with the default knot locations, we plotted the log cumulative hazard resulting from the WinBUGS model with 1, 2, and 3 knots against log time alongside the Nelson‐Aalen estimate of the log cumulative hazard and its 95% confidence intervals. For each trial, we chose the model with the number of knots that showed the best agreement between the WinBUGS model and the Nelson‐Aalen estimate. This resulted in 34 trials with 2 knots and 3 trials with 1 knot. Knot locations were then tweaked where necessary to improve the agreement between the WinBUGS model and the Nelson‐Aalen estimate and to ensure the log cumulative hazard from the WinBUGS model fell within the 95% confidence intervals of the Nelson‐Aalen estimate. A table of knot locations can be found in Appendix B.

Data formatting including the calculation of basis functions can be conducted in any statistical package. All models were run in WinBUGS^21^ version 1.4.3. The Stata suite of commands *winbugs* 27 was used to control all aspects of model fitting in WinBUGS through Stata^28^ version 14. Example Stata code for calculating basis functions and running WinBUGS from Stata along with the WinBUGS model is provided in Appendix A.

Initially, we considered fixed treatment effect (FTE) models with random treatment effect (RTE) models considered where there was evidence of heterogeneity. Final models were run with 20 000 burn‐in and then 20 000 iterations and with 2 sets of initial values. Convergence was checked by examining the trace and histograms of the posterior distribution. Models were compared using the deviance information criteria (DIC) statistic.^29^, ^30^

### 3.3 Results of pairwise MA using the Royston‐Parmar method

**Figure 2:**
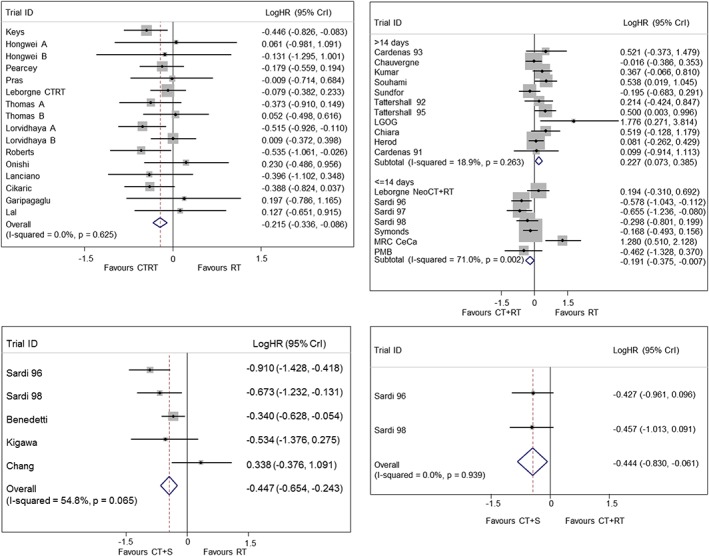
Pairwise fixed treatment effect meta‐analysis for all pairwise comparisons in the cervical cancer network. Top left: RT vs CTRT, top right: RT vs CT+RT, bottom left: RT vs CT+S, bottom right: CT+RT vs CT+S

Initially, we treated the network as 4 separate pairwise meta‐analyses and conducted a one‐step MA of each comparison using (2). Figure 2 shows 4 forest plots of log hazard ratios (LogHR) and 95% credible intervals (CrI). The resulting FTEs are presented in Table 1. The treatment effects were consistent with the treatment effects from a two‐stage pairwise MA using the Cox model. Results of the pairwise MA suggest CTRT improves overall survival by 19% compared to RT (LogHR=−0.215, 95% CrI: −0.336, −0.086), CT+S improves overall survival by 36% compared to RT (LogHR=−0.447, 95% CrI: −0.654, −0.243), and CT+S also improves overall survival by 36% compared to CT+RT (LogHR=−0.444, 95% CrI: −0.830, −0.061).

**Table 1:** Meta‐analysis results using Royston‐Parmar models

Comparison	FTE*	Cochran's Q	Global Non‐PH Test	Schoenfeld Residuals
RT vs CTRT	−0.215 (−0.336, −0.086)	12.71, 15 df, P=.625	χ ^2^=0.161, 1 df, P=.688	χ ^2^=25.64, 16 df, P=.059
RT vs CT+RT	−0.191 (−0.375, −0.007)	20.69, 6 df, P=.002	χ ^2^=2.522, 1 df, P=.112	χ ^2^=10.34, 7 df, P=.170
⩽14 days				
RT vs CT+RT	0.227 (0.073, 0.385)	12.34, 10 df, P=.263	χ ^2^=0.006, 1 df, P=.944	χ ^2^=7.65, 11 df, P=.744
>14 days				
RT vs CT+S	−0.447 (−0.654, ‐0.243)	8.85, 4 df, P=.065	χ ^2^=0.118, 1 df, P=.731	χ ^2^=8.65, 5 df, P=.124
CT+RT vs CT+S	−0.444 (−0.830, −0.061)	0.01, 1 df, P=.939	χ ^2^=0.164, 1 df, P=.686	χ ^2^=0.49, 2 df, P=.783

Abbreviations: CTRT, chemoradiation; CT+RT, neoadjuvant chemotherapy plus radiotherapy; CT+S, neoadjuvant chemotherapy plus surgery; FTE, fixed treatment effect; RT, radiotherapy; PH, proportional hazard.* Values are log hazard ratios and 95% credible intervals.

Cochran's Q statistic can be used to assess heterogeneity within each treatment comparison.^31^ There was no evidence of statistical heterogeneity within the RT vs CTRT (P=.625, Table 1), RT vs CT+S (P=.065), and CT+RT vs CT+S (P=.939) comparisons while there was some evidence of statistical heterogeneity in the RT vs CT+RT comparison (P<.001, also noted in the original publication^23^). When we split the RT vs CT+RT comparison into subgroups based on length of chemotherapy cycles, we found no evidence of heterogeneity in the trials with chemotherapy cycles greater than 14 days (P=.263). However, there was evidence of heterogeneity in the trials with chemotherapy cycle lengths of 14 days or less (P=.002). Heterogeneity can also be assessed visually by considering the forest plots in Figure 2. Due to the presence of heterogeneity in one of the pairwise comparisons going forward, we will need to consider RTE NMA models. There was no evidence globally of non‐PH in any of the treatment comparisons (Table 1, column 4); however, the Schoenfeld residuals indicate that there may be some trials in the RT vs CTRT comparison, which are at risk of non‐PH (P=.059, Table 1, column 5). However, we have performed multiple tests, and this is only borderline significant. Moreover, the global test of nonproportionality in log(t) is far from significant; therefore (in the light of our discussion at the end of Section 3.1), we continue under the assumption of PH in the cervical cancer network.

## 4 NETWORK META‐ANALYSIS USING ROYSTON‐PARMAR METHOD

### 4.1 One‐step IPD NMA model for time‐to‐event data

The one‐step NMA model models the log cumulative hazard individually for each trial with its own spline function (1) and location of knots. For patient i in trial j in a network of q+1 treatments, the FTE model takes the following form:

(6) $$\ln\{H_{j}(t|x_{ij})\}=s_{j}(\ln(t_{i}))+\beta_{1}{\text{trt1}}_{i}+\text{⋯}+\beta_{q}{\text{trtq}}_{i},$$

where trtq_i_ is a treatment contrast variable. Some care is needed in defining the treatment contrasts to ensure that they are in the right direction. This is necessary for the model to be properly defined. The treatment contrasts are patient level variables, which can take the value 0, 1, or −1. Where there are treatment loops in the network, the treatment contrasts represent the consistency equations. For example, in a 3‐treatment network consisting of treatments A, B, and C, where μ_AB_ is the treatment effect of treatment B compared to treatment A, the treatment effect for treatment C compared to treatment B can be calculated as μ_BC_=μ_AC_−μ_AB_. This means that only 2 treatment contrast variables (representing the coefficients of μ_AB_ and μ_AC_) need defining.

Specifically, in the cervical cancer network where there are 4 treatments (with one 3‐treatment loop, Figure 1), we need to define 3 treatment contrast variables. We chose to define the treatment contrast variables for RT vs CTRT, RT vs CT+RT, and RT vs CT+S. In Figure 1, the arrows indicate the direction of the treatment effects. RT is the reference treatment for trials comparing RT and CTRT, RT and CT+RT, and RT and CT+S. For trials comparing CT+RT and CT+S, CT+RT is the reference treatment and the treatment contrasts need to reflect this. For patients in a CT+RT vs CT+S trial receiving CT+S there must be a “−1” for the coefficient of RT vs CT+RT and a “1” for the coefficient of RT vs CT+S. For patients in a CT+RT vs CT+S trial receiving CT+RT, the coefficients of RT vs CT+RT and RT vs CT+S must both be “0.”

In other words, if trt1_i_ is the treatment contrast variable for RT vs CTRT, trt2_i_ is the treatment contrast variable for RT vs CT+RT, and trt3_i_ is the treatment contrast variable for RT vs CT+S, then

$$\begin{array}{ccc} {\text{trt1}}_{i} & =\left\{\begin{array}{cc} 1 & \text{if patient was randomised to CTRT and is from a trial comparing}\\ & \text{RT and CTRT}\\ 0 & \text{otherwise} \end{array}\right. & \\ {\text{trt2}}_{i} & =\left\{\begin{array}{cc} 1 & \text{if patient was randomised to CT+RT and is from a trial comparing}\\ & \text{RT and CT+RT}\\ -1 & \text{if patient was randomised to CT+S and is from a trial comparing}\\ & \text{CT+RT and CT+S}\\ 0 & \text{otherwise} \end{array}\right.\\ {\text{trt3}}_{i} & =\left\{\begin{array}{cc} 1 & \text{if patient was randomised to CT+S and is from a trial comparing}\\ & \text{RT and CT+S or CT+RT and CT+S}\\ 0 & \text{otherwise}. \end{array}\right. \end{array}$$

The corresponding RTE model takes the form:

(7) $$\ln\{H_{j}(t|x_{ij})\}=s_{j}(\ln(t_{i}))+\beta_{1j}{\text{trt1}}_{i}+\text{⋯}+\beta_{qj}{\text{trtq}}_{i}$$

$$\left(\begin{array}{c} \beta_{1}\\ \vdots \\ \beta_{q} \end{array}\right)∼MVN(\mu ,T),$$

where **T** is the unstructured inverse between‐study variance‐covariance matrix. In this paper, we use an unstructured covariance matrix because the cervical cancer network is a simple network with lots of data, which can support the estimation of an unstructured covariance matrix. Unless there is a strong a priori reason for a common heterogeneity variance, this is more plausible. However, when there are fewer trials, a simpler approach such as the Higgins and Whitehead^32^ approach to estimating the between‐study variance‐covariance matrix could also be used. This approach requires the estimation of only one parameter, and so is particularly popular when there is relatively little information available to estimate an unstructured covariance matrix.

#### 4.1.1 Global test for non‐PHs

We now detail 2 approaches for testing the assumption of PH. Firstly, a network test for non‐PH can be conducted by including an interaction between treatment and ln(time) in a FTE or RTE model:

(8) $$\begin{array}{ccc} \ln\{H_{j}(t|x_{ij})\} & =s_{j}(\ln(t_{i}))+\beta_{1j}{\text{trt1}}_{i}+\text{⋯}+\beta_{qj}{\text{trtq}}_{i} & \\ & \;+\beta_{(q+1)j}{\text{trt1}}_{i}\left(\ln(t_{i})\right)+\text{⋯}+\beta_{(2q)j}{\text{trtq}}_{i}\left(\ln(t_{i})\right), \end{array}$$

As before (Section 3.1.1), the derivative (3) of the log cumulative hazard must also be updated. Annotated model code based on the cervical cancer network in Figure 1 is provided in Appendix A. After fitting the model, we can perform an approximate global Wald test on the treatment‐ln(time) interaction terms to determine whether there is, on average, any evidence of non‐PH within the network. The null hypothesis states that the treatment‐ln(time) interactions are simultaneously equal to zero so that there is no evidence of non‐PH in the network. Details for conducting a Wald test can be found in Appendix C.

Our second approach that gives more insight into which trials are driving any nonproportionality is to allow the interaction terms to vary by trial. We can extend the FTE model (6) in this way:

(9) $$\begin{array}{ccc} \ln\{H_{j}(t|x_{ij})\} & =s_{j}(\ln(t_{i}))+\beta_{1j}{\text{trt1}}_{i}+\text{⋯}+\beta_{qj}{\text{trtq}}_{i} & \\ & \;+\left(\beta_{(q+1)j}+u_{j}\right){\text{trt1}}_{i}\left(\ln(t_{i})\right)+\text{⋯}\\ & \;+\left(\beta_{(2q)j}+u_{j}\right){\text{trtq}}_{i}\left(\ln(t_{i})\right) \end{array}$$

$$u_{j}∼N\left(0,\sigma_{u}^{2}\right),$$

Annotated model code based on the cervical cancer network in Figure 1 is provided in Appendix A. As before, an approximate global Wald test of the fixed treatment‐ln(time) and variance parameters can then be conducted to determine whether there is any evidence of non‐PH within the network. By allowing a random effect of treatment‐ln(time) by trial, we obtain a shrinkage estimate of the departures from PH in each trial. We can display this graphically by plotting the values of the u_j_ parameters along with an interval of u_j_ ±1.96sd_j_, where sd_j_ is the standard deviation of u_j_ for trial j.

Non‐PH in some or all of the trials can be accommodated by re‐fitting (8) or (9) and restricting the treatment‐ln(time) interaction terms to apply only to the trials exhibiting evidence of non‐PH. The timescale could then be divided up and the log hazard ratios assessed within each time interval. Alternatively, a spline that allows the treatment effect to vary over time could be added.

### 4.2 Assessment of inconsistency

A network is considered to be consistent when the treatment effect estimates from the direct comparisons are in agreement with the treatment effect estimates from the indirect comparisons. Therefore, inconsistency occurs within a treatment loop when the indirect evidence is not in agreement with the direct evidence. As a result, inconsistency is a property of a treatment loop not of a treatment comparison.^33^ This is different to heterogeneity, which can be defined as the amount of disagreement between trial‐specific treatment effects amongst trials comparing the same treatments.^34^

To assess inconsistency, we introduced a fixed effect inconsistency parameter to (6) following the method of Lu and Ades.^11^ This allowed us to obtain estimates of the direct and indirect information for each comparison within the treatment loop formed by RT, CT+RT, and CT+S. In a network containing one 3‐treatment loop between treatments A, B, and C, let ω_ABC_ represent the inconsistency parameter for this loop. We can then extend (6) in this way:

(10) $$\begin{array}{ccc} \ln\{H_{j}(t|x_{ij})\} & =s_{j}(\ln(t_{i}))+\beta_{1}{\text{trt1}}_{ij} & \\ & \;+\beta_{2}{\text{trt2}}_{ij}-\omega_{ABC}{\text{trt1}}_{ij}{\text{trt2}}_{ij}, \end{array}$$

An inconsistency parameter can be added to the RTE model in the same way. In passing, the inclusion of an inconsistency parameter allows us to test for inconsistency between two‐arm trials only as by definition multiarm trials are internally consistent. Note, we only need to fit one model with the inconsistency parameter to separate out the direct and indirect evidence for all trials in the loop. The cervical cancer network contains one treatment loop so only one inconsistency parameter was included in the model. See annotated model code in Appendix A for cervical cancer network shown in Figure 1. As noted in Section 4.4, similar results could be obtained by back‐calculation. For more complex networks, our models could readily be extended to incorporate node‐splitting.^35^

### 4.3 Inconsistency and heterogeneity

We briefly consider how to proceed if there is some evidence of heterogeneity (when we do not model inconsistency) and inconsistency (when we do not model heterogeneity, ie, in the FTE model).

First, suppose there is no funnel plot asymmetry in the pairwise MAs within the network so that the FTE point estimate and RTE point estimate are virtually identical. In this case, if there is inconsistency, then the extent of inconsistency will be the same in both the FTE and RTE model. However, if there is heterogeneity, then the standard error of the point estimate will be (appropriately) larger in the RTE than the FTE estimate. This will reduce the power to detect inconsistency in the RTE model.

Alongside, this is the fact that even in the FTE model, the inconsistency test has relatively low power. Therefore, in practice, if we find inconsistency in the FTE model, but there is also heterogeneity, then moving to a RTE model may mean the inconsistency is no longer detectable.

In practice, we would lean to the following approach: (1) fit the FTE model, test for inconsistency, and include an inconsistency parameter if needed; (2) fit the RTE model if needed (retain the inconsistency parameter if it was needed in the FTE model); (3) if the RTE model is needed, explore whether the conclusions are sensitive to including the inconsistency parameter (regardless of its formal significance); if they are, we would prefer to retain it. This approach could usefully be complemented by an initial assessment of heterogeneity in pairwise MA. If inconsistency is present, results should not be used for clinical inference without resolving the cause of the underlying inconsistency/heterogeneity.

### 4.4 Assessment of heterogeneity

Heterogeneity should be assessed within each pairwise comparison before an NMA model is fitted, both visually through the use of forest plots and using formal statistical tests. For the cervical cancer network, this was reported in Section 3.3. Once an FTE NMA model is fitted, Cochran's *Q* statistic can be used to assess heterogeneity within the network. The overall *Q* statistic from the FTE NMA model can be decomposed into within‐design heterogeneity (*Q* ^het^) and between‐design heterogeneity representing inconsistency between designs (*Q* ^inc^). Let ${\hat{\theta }}_{ij}$ be the treatment effect estimate for trial *i* of design *j*, ${\hat{\theta }}_{j}$ be the treatment effect from the direct evidence for design *j* only, and ${\hat{\theta }}_{Nj}$ be the network estimate of the treatment effect for design *j*, then

$$Q=\sum_{j}\sum_{i}{\left\{\frac{{\hat{\theta }}_{ij}-{\hat{\theta }}_{Nj}}{{\hat{\sigma }}_{ij}}\right\}}^{2}$$

$$Q^{\text{inc}}=\sum_{j}{\left\{\frac{{\hat{\theta }}_{j}-{\hat{\theta }}_{Nj}}{{\hat{\sigma }}_{j}}\right\}}^{2}$$

$$Q^{\text{het}}=\sum_{j}\sum_{i}{\left\{\frac{{\hat{\theta }}_{ij}-{\hat{\theta }}_{j}}{{\hat{\sigma }}_{ij}}\right\}}^{2},$$

with *Q*=*Q* ^inc^+*Q* ^het^. A corresponding matrix decomposition holds for multiarm trials. An alternative method of assessing heterogeneity would be to present values of *τ* ^2^.

### 4.5 Ranking of treatments

To rank the treatments, we took each iteration in turn and ranked the treatments from most effective to least effective. The most effective treatment had the smallest log hazard ratio value, and the least effective treatment had the largest log hazard ratio value. We then counted how many times each treatment was considered the first, second, third, fourth, and fifth most effective treatment and expressed these as percentages.

### 4.6 Prior distributions

In the FTE model, parameters representing the spline function for the baseline log cumulative hazard function, treatment effects, inconsistency parameters, and treatment‐ln(time) interactions were fitted with noninformative normal prior distributions (*γ*∼*N*(0,10000),*β*∼*N*(0,1000),*ω*∼*N*(0,10)). For model (9), *σ*_*u*_∼*N*(0,1000), which was restricted to be positive.

In the RTE model *β*∼*M* *V* *N*(*μ*,*T*) with *μ*∼(0,*σ*) and *σ* a matrix with 0.001 on the diagonal and 0 elsewhere. The prior distribution for *T* is an inverse Wishart distribution *T*∼*I* *W*(*V*,*k*) where V is a *p*x*p* scale matrix with the degrees of freedom, *k*(⩾*p*), as small as possible to reflect vague prior knowledge. Prior distributions for all other parameters remain the same as for the FTE model.

## 5 RESULTS

Here, we present the results of using the one‐step IPD Royston‐Parmar approach for NMA with the cervical cancer dataset introduced in Section 2. Parameter estimates are presented as log hazard ratios with 95% credible intervals for the posterior mean. A log hazard ratio of 0 indicates a null effect. A log hazard ratio less than zero indicates a beneficial effect relative to the reference treatment. In Section 3.3, we identified heterogeneity in the RT vs CT+RT comparison and presented results with the trials split by chemotherapy cycle length. In this section, the NMA model includes an additional parameter for cycle length, which, through the use of an indicator variable, can only contribute to the hazard in trials with long chemotherapy cycles. By doing this, we treat CT+RT short cycles and CT+RT long cycles as 2 separate treatments, so explaining this source of heterogeneity. Due to the presence of heterogeneity in one of the pairwise comparisons, we will consider both FTE and RTE models. The RTE model will take into account statistical heterogeneity.

Prior to conducting the NMA, to check that an appropriate spline model was chosen for each trial, the log cumulative hazard was fitted individually for each trial in WinBUGS and then plotted against the nonparametric Nelson‐Aalen estimate to assess the fit of the model to the data. All trials, except 3, used spline models with 2 interior knots, the remaining 3 trials used 1 interior knot.

We start by fitting FTE and RTE models in Section 5.1 and assessing the assumptions of PH (Section 5.1.1) and inconsistency (Section 5.2). In Section 5.3, we assess the network for any evidence of heterogeneity before ranking the treatments in order of effectiveness (Section 5.4).

### 5.1 Model results

**Figure 3:**
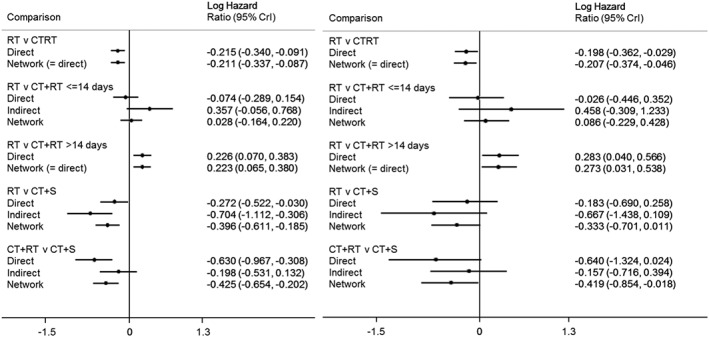
Cervical cancer results. Left: fixed treatment effect, right: random treatment effect

Figure 3 shows the direct, indirect, and network treatment effects for the cervical cancer network. The direct and indirect treatment effects are estimated through the inclusion of an inconsistency parameter as described in Section 4.2. We see that in this network, we have limited indirect evidence so that our network treatment effects are fairly close to the direct effects. Assuming consistency, the network treatment effect for CTRT compared to RT is statistically significant in both the FTE and RTE model with the RTE model suggesting an 18% improvement in overall survival with CTRT (LogHR=−0.207, 95% CrI: −0.374, −0.046, Table 2). The results of the FTE and RTE models are consistent with each other. The DIC provides only weak evidence in favour of the RTE model (difference in DIC of 5, Table 2); however, the presence of heterogeneity suggests that the RTE model is the best choice.

**Table 2:** Results of the fixed treatment effect (FTE) and random treatment effect (RTE) NMA models and DIC

FTE				RTE			
**Treatment Effects**		**Model Fit**		**Treatment Effects**		**Model Fit**	
RT	0	pD	138.6	RT	0	pD	152.2
CTRT	−0.211 (−0.337, −0.087)	$\bar{D}$	12182.9	CTRT	−0.207 (−0.374, −0.046)	$\bar{D}$	12163.6
CT+RT	0.028 (‐0.164, 0.220)	DIC	12321.5	CT+RT	0.086 (‐0.229, 0.428)	DIC	12315.8
⩽14 days				⩽14 days			
CT+RT	0.223 (0.065, 0.380)			CT+RT	0.273 (0.031, 0.538)		
>14 days				>14 days			
CT+S	−0.396 (‐0.611, −0.185)			CT+S	−0.333 (−0.701, 0.011)		

Abbreviations: CTRT, chemoradiation; CT+RT, neoadjuvant chemotherapy plus radiotherapy; CT+S, neoadjuvant chemotherapy plus surgery; DIC, deviance information criteria; NMA, network meta‐analysis; RT, radiotherapy.Results for treatment effects are log hazard ratios (95% credible intervals).

#### 5.1.1 Global test for non‐PHs

Here, we present the results from our 2 methods for assessing the assumption of PH. From the first approach, the Wald test for non‐PH from the RTE model with random treatment‐ln(time) interactions gave *χ* ^2^=0.324 on 3 degrees of freedom (*P*=.955) suggesting that, on average, there is no evidence of non‐PH within the network.

**Figure 4:**
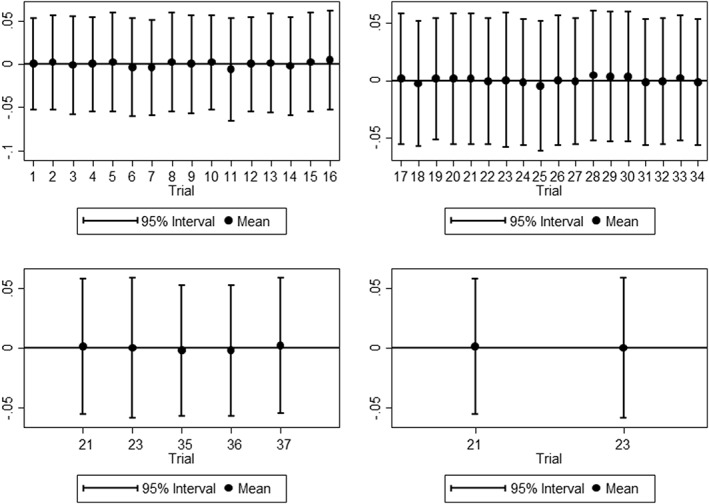
Variation in treatment‐ln(time) interactions for assessment of nonproportional hazard in random treatment effect network meta‐analysis model. Top left: RT vs CTRT, top right: RT vs CT+RT, bottom left: RT vs CT+S, bottom right: CT+RT vs CT+S

In the second approach, when we allow the treatment‐ln(time) interaction parameters to vary by trial, the Wald test for the RTE model gave *χ* ^2^=0.663 on 4 degrees of freedom (*P*=.956) suggesting that, on average, there is no evidence of non‐PH within the network. Figure 4 displays the amount of variation in the treatment‐ln(time) interactions for each trial from the RTE model with random treatment‐ln(time) interactions. There is little variation between trials supporting the conclusion, from the Wald test, that there is no evidence of non‐PH within the network.

### 5.2 Assessment of inconsistency

To assess inconsistency and to obtain estimates of the direct and indirect information for each comparison within the treatment loop, a fixed effect inconsistency parameter was introduced to the treatment loop formed by RT, CT+RT, and CT+S, as described in Section 4.2 and (10). From the RTE model, the inconsistency parameter was estimated as −0.484 (95% CrI: −1.314, 0.354). In Figure 3, we separate out the direct and indirect evidence for each treatment comparison and display these alongside the network estimates. It can be seen that the direct and indirect treatment effects differ from each other with the network estimates balancing out these 2 sources of information. Therefore, the cervical cancer network has a suggestion of inconsistency and the model results should be cautiously interpreted.

### 5.3 Assessment of heterogeneity

From the FTE model, there was evidence of statistically significant heterogeneity in the whole network (Q=56.86 on 35 df, P=.011) and between designs (Q=10.32, 2 df, P=.006). There was also some evidence of heterogeneity within each design (Q=46.21 on 33 df, P=.063), which was largely driven by the heterogeneity within the RT vs CT+RT (chemotherapy cycles less than 14 days) comparison (Q=16.74, 6 df, P=.010), as previously identified in Figure 2. The heterogeneity between designs was driven by the Sardi 96 trial.^36^ Sensitivity analysis excluding the Sardi 96 trial reduced the overall Q to borderline significance (Q=47.98 on 33 df, P=.044) and removed the inconsistency between designs (Q=2.53 on 2 df, P=.282). Treatment effect estimates for RT vs CT+RT with chemotherapy cycles less than or equal to 14 days and RT vs CT+S were slightly reduced in both the FTE and RTE models and remained consistent with each other.

### 5.4 Ranking of treatments

**Figure 5:**
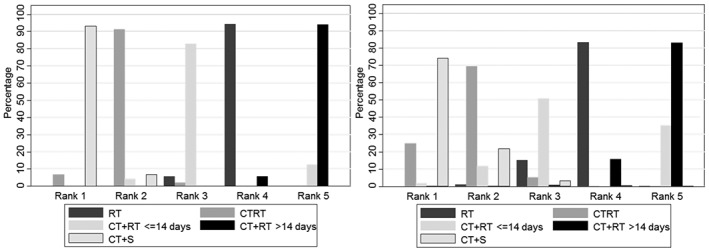
Treatment ranks from fixed treatment effect NMA model (left) and random treatment effect NMA model (right). NMA, network meta‐analysis

The ranking of treatments in order of most effective to least effective is consistent between the FTE and RTE models. In both models, CT+S comes out as the most effective treatment, CTRT the second most effective treatment, CT+RT with chemotherapy cycles less than or equal to 14 days the third most effective treatment, RT the fourth most effective treatment and CT+RT with chemotherapy cycles greater than 14 days as the least effective treatment (Figure 5).

## 6 DISCUSSION

The literature for conducting NMA with time‐to‐event data is rather sparse. This paper extends work by Royston and Parmar^20^ to the NMA setting, showing that Royston‐Parmar models, fitted in WinBUGS, provide a flexible, practical approach for Bayesian NMA with time‐to‐event data. They avoid the computational issues that beset a Bayesian implementation of the Cox model, which (see Section 1) we found computationally intractable for our cervical cancer network. An advantage of this approach is that, if we wish, we can readily obtain an estimate of the baseline hazard, pooled across trials. To do this, we make the coefficients for the RCS random across trials (this requires the knots to be in the same position for all studies). The Bayesian approach also provides a computationally straightforward and inferentially natural framework for ranking treatments.

The proposed approach naturally allows the inclusion of patient level covariates. The Bayesian aspect means we can readily allow covariates to have random coefficients, avoiding the numerical integration needed to maximize the corresponding likelihoods. This in turn naturally allows us to test for, and accommodate, departures from proportionality in some or all of the studies, by including appropriate treatment‐ln(time) interactions. Making these random (as in Equation 9) gives us a Bayesian shrinkage estimate of the extent of each study's departure from PH (Figure 4). The shrinkage reduces the likelihood of overinterpreting apparent departures from proportionality in smaller studies. Where proportionality is not appropriate, it naturally allows for—for example—effect estimation using restricted mean survival time as an estimate of treatment efficacy,^37^ which has so far been considered only in the MA setting.^38^

Network meta‐analysis combines direct and indirect evidence. Since the latter requires much stronger assumptions, it is sensible to check that they are consistent. We illustrated how this may be done using the model‐based version of the method proposed by Bucher.^33^ One inconsistency parameter is required for each treatment loop within a network, and we simply refit the NMA model with all these parameters included. This allows us to separate the direct and indirect contributions to each treatment effect (Figure 3). We believe these should always be presented, because readers should be aware of the extent to which conclusions rest on indirect evidence, with its attendant additional assumptions.

Besides the Cox model (discussed in Section 1), another option is a piecewise constant hazard model, also referred to as a piecewise Poisson model. With this model, the dataset needs to be expanded for each piecewise constant hazard. Thus, this approach is affected by the same issue as the Cox model, especially if a large number of intervals of piecewise constant hazard are required. Crowther^14^ suggested alleviating the computational burden this causes by collapsing across covariate patterns; however, this is not ideal and not possible with continuous covariates. By contrast, as our code shows, the Royston‐Parmar model avoids these issues. Nevertheless, there is a price to be paid in computational time. Where the same model can be fitted using a generic Bayesian program such as WinBUGS, and by maximum likelihood, WinBUGS will typically be slower than the corresponding, model specific, maximum likelihood software. However, this drawback is far from prohibitive. On a laptop with an Intel Core i7‐3540M processor with 4Gb of RAM, Model (6) took 0.045 second per update, so a burn in of 1000 updates followed by 4000 further updates to estimate the posterior takes less than 4 minutes.

It is also possible to conduct an IPD NMA using the Royston‐Parmar model as a two‐step approach and to fit the Royston‐Parmar model in the frequentist setting. In a two‐step approach, the Royston‐Parmar model is fitted individually to each trial and then study estimates of the log hazard ratio and its standard error can be pooled together in the second step. In the same way, a two‐step approach could be used with the Cox model. Indeed, we found the results of the one‐step FTE Royston‐Parmar MA model fitted in the Bayesian setting were consistent with the two‐step approach using the Cox model fitted in the frequentist setting for all 4 treatment comparisons in the cervical cancer network.

In the frequentist setting, the Royston‐Parmar model can be fitted in Stata using the *stpm2* 24 command and in R using the *flexsurv* 39 package. Two‐step IPD MA, using the Royston‐Parmar model or the Cox model, can be conducted in Stata using the *ipdmetan* 40 command. A random effects MA using the Royston‐Parmar model could be fitted in the frequentist setting using the Stata command *stmixed* 8 or using SAS PROC NLMIXED. However, both rely on numerical integration, which—as discussed in the Introduction—has some drawbacks.

This paper provides a base for further extensions. Work is currently ongoing to extend the Royston‐Parmar model to include covariates and treatment‐covariate interactions. A one‐stage Bayesian approach to fitting these models has many benefits as the models increase in complexity. This includes the ability to handle missing patient level covariates as part of the modelling. However, estimating treatment‐covariate interactions in an NMA needs to be done with care. We need to decide whether to model the covariate with trial specific or arm specific coefficients and need to separate out the within study and across network information, which is at risk of ecological bias.^41^

The NMA literature contains many examples when we wish to synthesize IPD and aggregate data. For example, Donegan^42^ showed how to combine IPD and aggregate data for dichotomous endpoints. Saramago^43^ showed how to do this in an FTE NMA model under the assumption that event times are Weibull distributed. In both cases, covariates can be included, with patient level values used for IPD trials and trial mean values used for aggregate data trials; however, PH can only be assessed in IPD trials. Synthesis of IPD and aggregate data is particularly natural in the Bayesian framework, where random effects can be naturally included to accommodate the inevitable heterogeneity. Therefore, the approach proposed here provides a flexible method of synthesising IPD and aggregate data for time‐to‐event outcomes, which avoids distributional assumptions.

We fitted our RTE models using an inverse Wishart prior for the between‐study variance‐covariance matrix. It has been highlighted by Wei and Burke that a Wishart prior may not be the most appropriate choice of prior distribution.^44^, ^45^ However, in the NMA setting where we have multiple treatments, there are few alternatives. A Wishart prior can become influential in the estimation of the between‐study variance‐covariance matrix and can lead to the overestimation of heterogeneity parameters particularly when the true heterogeneity is close to zero.^44^ Conducting NMA in the Bayesian framework allows for the possibility of including empirical evidence in the prior distributions, which could result in a more realistic prior distribution for the between‐study variance‐covariance matrix particularly when small numbers of trials are available.^46^

Network meta‐analysis models play a key role in policy decisions. Yet they are complex, both in terms of assumptions and modelling. We have found the following diagnostics useful:

1. using the shrinkage estimator to test for PH: the shrinkage reduces the likelihood of overinterpreting departures from PH;
2. graphically comparing the NMA spline estimate of the log cumulative hazard with the Nelson‐Aalen nonparametric estimate;
3. fitting a version of the model with an inconsistency parameter in each of the network loops, and using the results to present the direct, indirect, and combined treatment estimates;
4. using the Q statistics to identify heterogeneity. This may be addressed by including random effects in some trial comparisons or by conducting sensitivity analysis in which trials whose treatment effects diverge from the norm are excluded.

In summary, Bayesian NMA of IPD offers many practical advantages but is computationally problematic with the Cox PH model, even with moderate size datasets. We have shown that the Royston‐Parmar model provides a flexible, computationally practical, way forward which has the potential to extend to accommodate issues such as non‐PH which are increasingly arising in oncology studies.

## Supporting information

Supporting info item

Supporting info item
