# Supplementary material for: Bayesian one‐step IPD network meta‐analysis of time‐to‐event data using Royston‐Parmar models
Source: Res Synth Methods. 2017 Jul 25;8(4):451–64. doi: 10.1002/jrsm.1253 (PMC5724680; doi:10.1002/jrsm.1253)
Supplement: Supplementary file 2 — Supporting info item [file JRSM-8-451-s002.pdf]

Bayesian one-step IPD network meta-analysis of time-to-event data using Royston-Parmar models: Supplementary material

S. C. Freeman, J. R. Carpenter

A. Worked example using the cervical cancer network

Treatment parameterisation

For the cervical cancer network, let trt1_i be the indicator variable for RT v CTRT, trt2_i be the indicator variable for RT v CT+RT and trt3_i be the indicator variable for RT v CT+S. The treatment contrast variables are defined as:

$$\text{trt1}_i = \begin{cases} 1 & \text{if patient was randomised to CTRT and is from a trial comparing} \\ & \text{RT and CTRT} \\ 0 & \text{otherwise} \end{cases}$$

$$\text{trt2}_i = \begin{cases} 1 & \text{if patient was randomised to CT+RT and is from a trial comparing} \\ & \text{RT and CT+RT} \\ -1 & \text{if patient was randomised to CT+S and is from a trial comparing} \\ & \text{CT+RT and CT+S} \\ 0 & \text{otherwise} \end{cases}$$

$$\text{trt3}_i = \begin{cases} 1 & \text{if patient was randomised to CT+S and is from a trial comparing} \\ & \text{RT and CT+S or CT+RT and CT+S} \\ 0 & \text{otherwise} \end{cases}$$

Stata code for initial data processing

In this subsection we present Stata code for the initial data processing steps which includes loading data, setting data as survival data and generating the treatment contrast variables.

```
*** Load data
cd $data
use alltrials, clear

*** Set data as survival data
nois stset t_os, scale(365.25) fail(surv==1)

gen t = _t
gen d = _d
gen lnt=ln(t)

*** Create a scalar and macro for number of trials
scalar Ntrials = 37

*** Create variable which distinguishes between different designs
* (design: 1=RT v CTRT, 2=RT v CT+RT, 3 =RT v CT+S, 4=CT+RT v CT+S)
gen design=1 if trialid<=16
replace design=2 if trialid>=17 & trialid<=32
replace design=3 if trialid>=35
replace design=4 if trialid==33 | trialid==34
```

```
*** Create treatment contrast variables
* (trt: 0=RT, 1=CTRT, 2=CT+RT, 3=CT+S)

gen trt1=.
gen trt2=.
gen trt3=.

replace trt1=0 if design==1
replace trt1=1 if trt==1 & design==1
replace trt2=0 if design==1
replace trt3=0 if design==1

replace trt2=0 if design==2
replace trt2=1 if trt==2 & design==2
replace trt1=0 if design==2
replace trt3=0 if design==2

replace trt3=0 if design==3
replace trt3=1 if trt==3 & design==3
replace trt1=0 if design==3
replace trt2=0 if design==3

replace trt1=0 if design==4
replace trt2=0 if trt==2 & design==4
replace trt3=0 if trt==2 & design==4
replace trt2=-1 if trt==3 & design==4
replace trt3=1 if trt==3 & design==4
replace trt2=0 if trt==0 & design==4
replace trt3=0 if trt==0 & design==4

*** In the cervical cancer network we split the RT v CT+RT comparison
*into two based on chemotherapy cycle length
```

```
*** Create variable for cycle length (1=long cycles)
gen cycles=1 if trt2==1 & (trialid==17 | trialid==18 | trialid==19
| trialid==24 | trialid==25 | trialid==27 | trialid==28 ///
| trialid==29 | trialid==32 | trialid==33 | trialid==34)
replace cycles=0 if cycles==.

*** Calculate trt*ln(time) interactions
*(for models including trt*ln(time) interactions only)
gen trt1lnt = trt1*lnt
gen trt2lnt = trt2*lnt
```

Calculating basis functions

In this subsection we explain how to calculate the basis functions introduced in Section 3.1.

For a patient i with survival (or censoring time) $ln(t_i)$ and a spline model with p interior knots where the location of the knots are $k_0, k_1, \ldots, k_p, k_{p+1} (k_0 < k_1 < ... < k_p < k_{p+1})$ and k_0 and k_{p+1} are the boundary knots and where $(x)_+$ equals x if $x > 0$ and 0 otherwise, the basis functions $v_0\big(ln(t_i)\big), v_1\big(ln(t_i)\big), v_2\big(ln(t_i)\big), \ldots, v_{p-1}\big(ln(t_i)\big), v_p\big(ln(t_i)\big)$ are calculated as follows:

$$v_0\big(ln(t_i)\big) = ln(t_i)$$

$$v_1\big(ln(t_i)\big) = \big(ln(t_i) - k_1\big)_+^3 - \phi_1\big(ln(t_i) - k_0\big)_+^3 - (1 - \phi_1)\big(ln(t_i) - k_{p+1}\big)_+^3, \text{ where } \phi_1 = \frac{(k_{p+1} - k_1)}{(k_{p+1} - k_0)}$$

$$v_2\big(ln(t_i)\big) = \big(ln(t_i) - k_2\big)_+^3 - \phi_2\big(ln(t_i) - k_0\big)_+^3 - (1 - \phi_2)\big(ln(t_i) - k_{p+1}\big)_+^3, \text{ where } \phi_2 = \frac{(k_{p+1} - k_2)}{(k_{p+1} - k_0)}$$

$$\vdots$$

$$v_{p-1}\big(ln(t_i)\big) = \big(ln(t_i) - k_{p-1}\big)_+^3 - \phi_{p-1}\big(ln(t_i) - k_0\big)_+^3 - (1 - \phi_{p-1})\big(ln(t_i) - k_{p+1}\big)_+^3, \text{ where } \phi_{p-1} = \frac{(k_{p+1} - k_{p-1})}{(k_{p+1} - k_0)}$$

$$v_p\big(ln(t_i)\big) = \big(ln(t_i) - k_p\big)_+^3 - \phi_p\big(ln(t_i) - k_0\big)_+^3 - (1 - \phi_p)\big(ln(t_i) - k_{p+1}\big)_+^3, \text{ where } \phi_p = \frac{(k_{p+1} - k_p)}{(k_{p+1} - k_0)}$$

Gram-Schmidt orthogonalisation is used to transform $v_0\big(ln(t_i)\big), v_1\big(ln(t_i)\big), \ldots, v_{p-1}\big(ln(t_i)\big), v_p\big(ln(t_i)\big)$ into $u_0\big(ln(t_i)\big), u_1\big(ln(t_i)\big), \ldots, u_{p-1}\big(ln(t_i)\big), u_p\big(ln(t_i)\big)$ which can then be used in equations (1), (2),

(3), (5), (6), (7), (8), (9) and (10). Let $u_0 = u_0\big(ln(t_i)\big)$, $u_1 = u_1\big(ln(t_i)\big)$, $un_1 = un_1\big(ln(t_i)\big)$ etc., then

$$u_0 = \frac{v_0 - mean(v_0)}{sd(v_0)}$$

$$un_1 = v_1 - \frac{< v_1, u_0 >}{< u_0, u_0 >}u_0, \text{ and normalising } u_1 = \frac{un_1 - mean(un_1)}{sd(un_1)}$$

$$un_2 = v_2 - \frac{< v_2, u_0 >}{< u_0, u_0 >}u_0 - \frac{< v_2, u_1 >}{< u_1, u_1 >}u_1, \text{ and normalising } u_2 = \frac{un_2 - mean(un_2)}{sd(un_2)}$$

$$\vdots$$

$$un_p = v_p - \frac{< v_p, u_0 >}{< u_0, u_0 >}u_0 - \frac{< v_p, u_1 >}{< u_1, u_1 >}u_1 - \cdots - \frac{< v_p, u_{p-1} >}{< u_{p-1}, u_{p-1} >}u_{p-1}, \text{ and normalising}$$

$$u_p = \frac{un_p - mean(un_p)}{sd(un_p)}$$

where $< u\big(ln(t_{ij})\big), v\big(ln(t_{ij})\big) >= \sum_i u\big(ln(t_{ij})\big)v\big(ln(t_{ij})\big)$ i.e. the inner product of the vectors u and v.

Stata code for calculating the basis functions

This subsection includes the Stata code for calculating the basis functions. We start by first specifying the location of the internal knots. In this example code we assume that all trials have the same two internal knot locations (based on percentiles of survival time). If the knot locations are chosen individually for each trial (as in the cervical cancer network) then these should be specified individually for each trial.

```
*** Set up location of knots for each trial
forvalues i=1(1)4 {
gen k`i'=.
}

*Here we assume all trials have the same two internal knot locations
*at the 33rd and 67th percentiles of uncensored survival times
forvalues i=1(1)37 {
```

```
centile lnt if status==1 & trialid==`i', centile(0 33 67 100)
replace k1 = `r(c_1)' if trialid==`i'
replace k2 = `r(c_2)' if trialid==`i'
replace k3 = `r(c_3)' if trialid==`i'
replace k4 = `r(c_4)' if trialid==`i'
}
```

Next, the orthogonalised basis functions are calculated for each trial in turn. To do this all the variables required for calculation of the basis functions are initially created as empty variables before being filled in.

```
*** First create empty variables which we will then fill below
gen u0=.
gen u1=.
gen u1n=.
gen u2=.
gen u2n=.
gen du0=.
gen du1=.
gen du1n=.
gen du2=.
gen du2n=.
gen phi1=.
gen phi2=.
gen v0=.
gen v1=.
gen v2=.
gen v1u0=.
gen u0u0=.
gen v2u0=.
gen v2u1n=.
gen u1nu1n=.
```

```
gen a=.
gen b=.
gen c=.
gen e=.
gen f=.
gen g=.
gen h=.
gen i=.
gen j=.
gen k=.
gen l=.
gen m=.
gen n=.

*** Orthogonalisation of 2 knots
* In this step we create the orthogonalised basis functions

forvalues i=1(1)37 {

replace a=max(lnt-k2, 0) if trialid==`i'
replace b=max(lnt-k1, 0) if trialid==`i'
replace c=max(lnt-k4, 0) if trialid==`i'
replace e=max(lnt-k3, 0) if trialid==`i'

replace phi1=(k4-k2)/(k4-k1) if trialid==`i'
replace phi2=(k4-k3)/(k4-k1) if trialid==`i'

replace v0 = lnt if trialid==`i'
replace v1 = a^3 - (phi1*(b^3)) - ((1-phi1)*(c^3)) if trialid==`i'
replace v2 = e^3 - (phi2*(b^3)) - ((1-phi2)*(c^3)) if trialid==`i'

*Calculate u0
```

```
su v0 if trialid==`i'
replace u0=(v0-`r(mean)')/(`r(sd)') if trialid==`i'

*Prepare to calculate u1
replace f=v1*u0 if trialid==`i'
replace g=u0*u0 if trialid==`i'
egen v1u0_t`i'=sum(f) if trialid==`i'
egen u0u0_t`i'=sum(g) if trialid==`i'

*Calculate u1 and then normalise
replace u1 = v1 - ((v1u0_t`i'/u0u0_t`i')*u0) if trialid==`i'
su u1 if trialid==`i'
replace u1n=(u1-`r(mean)')/(`r(sd)') if trialid==`i'

*Prepare to calculate u2
replace h=v2*u0 if trialid==`i'
egen v2u0_t`i'=sum(h) if trialid==`i'
replace i=v2*u1n if trialid==`i'
egen v2u1n_t`i'=sum(i) if trialid==`i'
replace j=u1n*u1n if trialid==`i'
egen u1nu1n_t`i'=sum(j) if trialid==`i'

*Calculate u2 and then normalise
replace u2 = v2 - ((v2u0_t`i'/u0u0_t`i')*u0) - ((v2u1n_t`i'/u1nu1n_t`i')*u1n) ///
if trialid==`i'
su u2 if trialid==`i'
replace u2n = (u2-`r(mean)')/(`r(sd)') if trialid==`i'

*Calculate deivative of u0
su v0 if trialid==`i'
replace du0=1/(`r(sd)') if trialid==`i'
```

```
*Calculate derivative of u1n
replace k = (3*(a^2)) - ((3*phi1)*(b^2)) - (3*(1-phi1)*(c^2)) if trialid==`i'

su u1 if trialid==`i'
replace l = `r(sd)' if trialid==`i'

replace du1n=(1/l)*k - (((v1u0_t`i'/u0u0_t`i')/l)*du0) if trialid==`i'

*Calculate derivative of u2n
replace m = (3*(e^2)) - ((3*phi2)*(b^2)) - (3*(1-phi2)*(c^2)) if trialid==`i'

su u2 if trialid==`i'
replace n=`r(sd)' if trialid==`i'

replace du2n=(1/n)*m - ((v2u0_t`i'/u0u0_t`i')/n)*du0 ///
- ((v2u1n_t`i'/u1nu1n_t`i')/n)*du1n if trialid==`i'

}
```

Stata code for running WinBUGS

In the next section of Stata code we use the *winbugs* suite of commands to prepare the data for WinBUGS, write a script file (which will tell WinBUGS which files to run), run the model in WinBUGS and load the results back in Stata.

```
*** Prepare data for WinBUGS

* First create a variable which starts with 0 and then includes the number
*of patients for each trial
gen offset = 0 if _n==1

forvalues i=1(1)37 {
```

```
count if trialid == `i'
replace offset = offset[`i']+`r(N)' if _n==`i'+1

}

*Create data file
*Include treatment contrast variables and basis functions
*d represents event indicator
*In cervical cancer network we also need the cycles variable
*offset identifies where one trial starts and ends
*Ntrials is the total number of trials in the network
wbdata, contents(wbvector d trt1 trt2 trt3 u0 u1n u2n du0 du1n du2n cycles, ///
format(%3.0f %3.0f %3.0f %3.0f %16.0g %16.0g %16.0g %16.0g ///
%16.0g %16.0g %3.0f) + ///
wbvector offset in 1/38, format(%2.0f) + ///
wbscalar, scalar(Ntrials) format(%2.0f)) ///
saving("data.txt",replace) noprint

* Write WinBUGS script file
wbscript, sav("script.txt", replace) /// Name the script file
model("`c(pwd)'/model.txt") /// Location of model file
data("`c(pwd)'/data.txt") /// Location of data file
inits("`c(pwd)'/inits.txt") /// Location of files for initial values
burn(20000) update(20000) ///  No. of MCMC iterations
set(gamma beta hr.trt1 hr.trt2 lhr.trt1vtrt2 hr.trt1vtrt2) ///
chains(2) ///
log("`c(pwd)'/log.txt") /// Where to put log file
codafile("`c(pwd)'/coda") /// Where to put CODA file
noprint dic ///
quit

* Run WinBUGS
```

```
wbrun, script("`c(pwd)'/script.txt") ///
win("C:\WinBUGS14\WinBUGS14")

* Load CODA output
wbcoda, root("`c(pwd)'/coda") chains(2) multichain clear

*Display results
wbstats*
```

WinBUGS model code

This next section specifies the one-step RTE NMA model (6) that was run in WinBUGS using the cervical cancer network. In the model i is patient and j is trial. This model specification also shows how the spline can be extended to fit models (8), (9) and (10). Note for these models the data file will need to be modified to include the treatment-ln(time) interactions or the indicator variable for the inconsistency parameter.

```
Model  {

for(j in 1:Ntrials) {

for(i in offset[j]+1:offset[j+1]) {

zeros[i] <- 0

# Spline
eta[i, j] <- gamma[1, j] + gamma[2, j]*u0[i] + gamma[3, j]*u1[i]
+ gamma[4, j]*u2[i]
# Covariates
+ beta[j, 1]*trt1[i] + beta[j, 2]*trt2[i] + beta[j, 3]*trt3[i]
```

```
+ beta[j, 4]*trt2[i]*cycles[i]

# Inconsistency parameter: Extension to (10)
# Consider the treatment loop in Figure 1 to be coded as:
# A = RT, B = CT+RT, C = CT+S
# - inconBC*trt2[i]*trt3[i]

# Extension to (7)
# Treatment-ln(time) interactions for assessment of non-proportional hazards
# + beta[j, 4]*trt1[i]*lnt[i] + beta[j, 5]*trt2[i]*lnt[i]
# + beta[j, 6]*trt3[i]*lnt[i]

# Extension to (8)
# Treatment-ln(time) interactions for assessment of non-proportional hazards
# allowing interaction terms to vary by trial
# + (beta[j, 4] + u[j])*trt1[i]*lnt[i] + (beta[j, 5] + u[j])*trt2[i]*lnt[i]
# + (beta[j, 6] + u[j])*trt3[i]*lnt[i]

# Derivative with respect to ln(t)
d.sp[i, j] <- gamma[2, j]*du0[i] + gamma[3, j]*du1[i] + gamma[4, j]*du2[i]

# Update derivative when including treatment-ln(time) interactions
# Extension to (7)
# + beta[4]*trt1[i] + beta[5]*trt2[i] + beta[6]*trt3[i]
# Extension to (8)
# + (beta[4]+u[j])*trt1[i] + (beta[5]+u[j])*trt2[i]
# + (beta[6]+u[j])*trt3[i]

# Likelihood
lnL[i] <- max( - ( d[i] * ( log( max( d.sp[i, j],0.0001 ) )
+ eta[i, j]- exp(eta[i, j]) ) - (1-d[i])*exp(eta[i, j]) ), 0.000001)
```

```
# Use zeros trick to maximise likelihood
zeros[i] ~ dpois(lnL[i])

}

# Prior Distributions

for(p in 1:4) {
gamma[p, j] ~ dnorm(0,0.0001)
}

# Priors for random coefficients:
      beta[j, 1:3 ] ~ dmnorm(mu[1:3 ], T[1:3 ,1:3 ])

}

# Hyper-priors:
   mu[1:3] ~ dmnorm(pmu[1:3 ], pT[1:3 ,1:3 ])
   T[1:3 ,1:3 ] ~ dwish(R[1:3 ,1:3 ], 3)

# inconBC ~ dnorm(0, 0.1)

# Convert log hazard ratios to hazard ratios
hr.trt1 <- exp(mu[1])
hr.trt2 <- exp(mu[2])
hr.trt3 <- exp(mu[3])

# Calculate direct effect for BC (Log Hazard Ratio)
# mu4 <- mu[3] - mu[2] + inconBC

# Indirect treatment effects (Log Hazard Ratios)
# thetaAB.ind <- mu[3] - mu4
```

```
# thetaAC.ind <- mu[2] + mu4
# thetaBC.ind <- mu[3] - mu[2]
}
```

B. Location of knots

Table 1: Knot locations from restricted cubic spline for each cervical cancer trial. Values are ln(time). Three trials had one interior knot only which is represented by n/a in the 2nd knot column.

Trial ID	Minimum	Percentile	1st Knot	Percentile	2nd Knot	Percentile	Maximum	Percentile
Keys	-0.96	0%	-0.5	5%	1	63%	2.41	100%
Hongwei A	-0.69	0%	-0.5	21%	1	86%	1.05	100%
Hongwei B	-0.02	0%	0.9	44%	1.1	57%	1.66	100%
Pearcey	-0.76	0%	0.2	32%	1	69%	2.67	100%
Pras	-0.92	0%	-0.2	11%	1	74%	1.84	100%
Leborgne CTRT	-3.60	0%	1.5	96%	n/a	n/a	2.05	100%
Thomas A	-2.77	0%	0.2	34%	1	75%	2.00	100%
Thomas B	-1.06	0%	0	35%	1.5	94%	1.77	100%
Lorvidhaya A	-3.13	0%	-1.5	6%	0.5	72%	1.81	100%
Lorvidhaya B	-2.40	0%	-0.1	34%	0.6	67%	2.04	100%
Roberts	-1.70	0%	0	35%	1	75%	1.95	100%
Onishi	-0.94	0%	-0.5	15%	1	65%	2.10	100%
Lanciano	-3.82	0%	-0.5	12%	1	70%	1.89	100%
Cikaric	-2.53	0%	-0.2	20%	0.8	80%	1.52	100%
Garipagaoglu	-0.52	0%	0.4	30%	1	67%	1.50	100%
Lal	-2.16	0%	-0.5	26%	0.8	92%	0.94	100%
Cardenas 93	-0.81	0%	1	78%	1.5	85%	2.09	100%
Chauvergne	-3.19	0%	-0.5	14%	0.5	51%	2.84	100%
Kumar	-1.77	0%	0	45%	1	83%	2.28	100%
Leborgne CT+RT	-2.14	0%	-1	6%	0	31%	1.99	100%
Sardi 96	-1.79	0%	-1	4%	1	88%	1.88	100%
Sardi 97	-0.26	0%	1	55%	1.5	83%	1.99	100%
Sardi 98	-0.09	0%	0.5	21%	1	53%	2.19	100%
Souhami	-2.03	0%	-0.5	30%	0.5	76%	1.26	100%
Sundfor	-2.81	0%	-0.5	10%	1	86%	1.78	100%
Symonds	-2.77	0%	-1	9%	1	82%	2.07	100%
Tattershall 92	-2.24	0%	0	39%	1	80%	1.34	100%
Tattershall 95	-3.34	0%	-1	12%	1	98%	1.02	100%
LGOG	-0.44	0%	0.6	68%	n/a	n/a	1.16	100%

Table 1: Knot locations from restricted cubic spline for each cervical cancer trial. Values are ln(time). Three trials had one interior knot only which is represented by n/a in the 2nd knot column.

Trial ID	Minimum	Percentile	1st Knot	Percentile	2nd Knot	Percentile	Maximum	Percentile
MRC CeCa	-0.53	0%	1	83%	1.5	94%	1.72	100%
PMB	-1.97	0%	1	85%	n/a	n/a	2.24	100%
Chiara	-0.97	0%	1	62%	1.5	81%	2.31	100%
Herod	-5.21	0%	1	79%	1.5	93%	2.13	100%
Cardenas 91	-0.57	0%	0	23%	1	83%	2.23	100%
Benedetti	-2.53	0%	-1	7%	1	81%	1.73	100%
Kigawa	-1.47	0%	-1	5%	1.5	85%	1.97	100%
Chang	-0.75	0%	-0.3	22%	0.5	60%	1.81	100%

C. Global Wald test

An approximate global Wald test on parameter estimates can be obtained by importing WinBUGS output into Stata and using the programming language Mata which is accessible through Stata. Let p be the number of parameters you wish to test and n be the number of iterations of the model performed in WinBUGS then the following algorithm can be used to conduct the Wald test:

1. Let the matrix B contain the results from WinBUGS of the n iterations for each interaction term. B will be a $n \times p$ matrix.
2. Calculate the mean value for each p and store as a vector called M. M will be a $1 \times p$ vector. Duplicate the row of mean values to get a $n \times p$ matrix in which each row is identical. This makes B and M matrices of the same dimension (which is necessary for the next step).
3. Calculate $C = B - M$. C will be a $n \times p$ matrix.
4. Calculate $A = \frac{C'C}{n}$. A will be a $p \times p$ matrix.
5. Take the column means of the matrix B and store as a vector called Z. Z will be a $1 \times p$ vector.
6. The Wald test is equal to Z multiplied by A multiplied by the transpose of Z ($\chi^2 = ZAZ'$).
7. Compare χ^2 to a distribution with p degrees of freedom.
